# Depression, anxiety and stress symptoms in Brazilian university students during the COVID-19 pandemic: Predictors and association with life satisfaction, psychological well-being and coping strategies

**DOI:** 10.1371/journal.pone.0258493

**Published:** 2021-10-13

**Authors:** Adriana Rezende Lopes, Oscar Kenji Nihei

**Affiliations:** Education, Languages and Health Center, Western Parana State University (UNIOESTE), Foz do Iguaçu, Parana, Brazil; National Cheng Kung University College of Medicine, TAIWAN

## Abstract

**Background:**

The COVID-19 pandemic raises concerns about the mental health of the world population. Protection measures to prevention the disease impacted education and undergraduate students were exposed to additional stressors.

**Objectives:**

Analyze depression, anxiety and stress symptoms in undergraduates, their respective predictors and the association with satisfaction with life, psychological well-being and coping strategies.

**Methods:**

An online cross-sectional study was conducted from September 14 to October 19, 2020, involving undergraduate students enrolled in 33 courses from 5 public university campuses in the state of Parana, Brazil, using: questionnaire with sociodemographic, academic, health and pandemic effects variables; Depression, Anxiety and Stress Scale-21 (DASS-21); Satisfaction with Life Scale (SWLS); Psychological Well-Being (PWB); BriefCOPE. The convenience sample was composed of 1,224 participants, with 18 years old or older, that completed all research instruments. Spearman correlation and logistic analysis (univariate and multivariate) were applied to the collected data.

**Results:**

Most of the undergraduates presented symptoms of depression (60.5%), anxiety (52.5%) and stress (57.5%). Depression, anxiety and stress presented significant correlations in common: negative with satisfaction with life, all dimensions of psychological well-being, and 3 adaptive copings (active coping, planning, positive reframing); positive with 5 maladaptive copings (behavioral disengagement, denial, self-blame, self-distraction, substance use). In addition, there were 7 common predictors for symptoms of depression, anxiety and stress: female; age 18–24 years old; having a chronic disease; lower scores in 2 dimensions of psychological well-being (positive relations with others, self-acceptance); higher scores in 2 maladaptive copings (self-blame, substance use).

**Conclusions:**

The data indicate a high prevalence of symptoms of depression, anxiety and stress, and suggest that higher scores of satisfaction with life, psychological well-being dimensions and adaptive copings may present protective effects in undergraduates during a pandemic crisis.

## Introduction

The crises generated by the COVID-19 pandemic have raised concerns about the mental health of the world population [1], notably in relation to the psychological impacts, already observed in previous pandemics, resulting from quarantine procedures (public health strategy recommended to stop the virus’ spread and prevent the collapse of health systems), in which sudden changes in daily routine are needed, such as suspension of in-person activities, adoption of distancing measures and social isolation, in addition to generating fear about the infection, severity of the disease and production of economic and social effects [2]. The strategies of social distancing, staying at home, mask wearing, and hand washing helped to reduce contamination with coronavirus [3].

The COVID-19 pandemic imposes obstacles, impediments and threats to physical and mental health, conditions to which the world population is being exposed and are considered to cause stress [4]. Studies have shown symptoms of depression, anxiety and stress in the population of different countries [5, 6]. In Brazil, studies indicate the presence of such symptoms [7–10], in addition to reports of frequent feelings of sadness/depression and nervousness/anxiety, in 40.4% and 52.6% of the Brazilian population, respectively [11].

A review study before the COVID-19 pandemic suggested that university students are vulnerable to stressors related to academic and relational life characteristics, likely to cause psychological distress [12]. Studies showed worrying percentages of stress among university students in Brazil (52.9% and 68.7%) before the pandemic [13, 14].

In the COVID-19 pandemic, undergraduates were exposed to additional stressors, as the disease prevention and protection measures impacted education, with interruption of in-person activities, change of schedules and transition of classes to virtual classrooms using the internet in most of the educational institutions around the world, leading to uncertainties regarding professional training and increasing levels of stress and anxiety [15, 16]. Furthermore, the COVID-19-related fear was associated with increased future career anxiety in Bangladesh university students [17, 18].

A study in the Spanish population during the COVID-19 pandemic showed that younger people (18–25 years old), mostly university students, reported more symptoms of depression, anxiety and stress, and the authors associated this result with the additional stress of the need to adapt to remote educational activities [19]. Undergraduate health care students undergoing clinical training may have increased stress and anxiety under the following conditions: high risk of being infected with coronavirus, fear of transmitting undiagnosed COVID-19 to others, lack of adequate personal protective equipment and resources, overload of responsibilities [20–22].

Study with Asian university students (Malaysia, Saudi Arabia, Pakistan, Bangladesh, China, India, Indonesia) observed that 35.6% experienced mild to extreme severe anxiety [23]. Other studies with undergraduates from Bangladesh, Egypt, Ethiopia, Lebanon and Turkey during the COVID-19 pandemic found wide variation in the percentages of symptoms of depression (21.2% to 76.1%), anxiety (27.7% to 71.5%) and stress (12.7% % to 70.1%) [24–30]. In Brazil, a study showed that 89.5% of university students in the health field reported they were distressed during the pandemic [31].

Comparing the periods before and during the COVID-19 pandemic, studies with university students showed: in Portugal, higher levels of depression, anxiety and stress in the pandemic period [32]; in Poland, an increase in the level of depression as the pandemic progresses [33]; in Italy, depressive symptoms worsened during the lockdown, but with its end, the symptoms levels were comparable to the ones reported before the COVID-19 pandemic [34]; in Brazil, a study in the state of São Paulo (Campinas municipality), did not detect a significant difference in perceived stress and symptoms of depression [35]. However, in the adult population in the state of Rio Grande do Sul (Brazil), a study that compared the period before and during the pandemic (June/July 2020) found that moderate to severe symptoms of depression and anxiety increased 6.6 and 7.4 times, respectively [36].

Psychological well-being (characteristic dimensions of healthy functioning [37]) and satisfaction with life (evaluation of one’s own personal situation, cognitive component of subjective well-being [38]), have been associated with the protection of the undergraduates’ mental health [39–42].

The adopted coping strategies, which are efforts to prevent or reduce threat, harm or loss, or to reduce the associated stress [4], may favor the adjustment to daily stressful situations and the physical and psychological well-being [43]. Studies suggest that the type of coping strategy adopted by undergraduates may be a risk or protective factor for psychological distress [12, 44] and psychological well-being [45, 46]. During the COVID-19 pandemic, studies observed that the effects of the pandemic crisis on the mental health of undergraduates can be reduced or increased according to the adopted coping strategies [20, 35, 47–49].

However, studies that addressed the levels and predictors of symptoms of depression, anxiety and stress, associating them with psychological well-being, satisfaction with life and coping strategies in university students during the COVID-19 pandemic are still scarce, and no similar study was found in Brazil, where the pandemic has a strong impact in terms of the number of deaths and in the social, economic and academic conditions of these undergraduate students.

During the research (September 14 to October 19, 2020), conducted in undergraduate students from the state of Parana, Brazil, the total number of deaths in the country increased from 133,006 to 154,175, and the number of accumulated cases increased from 4,345,610 to 5,250,727 [50]. The first confirmed case of COVID-19 in Brazil occurred in February 2020 and, in the state of Parana, on March 12, 2020, one day after the World Health Organization (WHO) declared the COVID-19 pandemic in the world [51].

The present study aimed to analyze the psychological impact of the COVID-19 pandemic on undergraduate students in Brazil, through the study of symptoms of depression, anxiety and stress, evaluating the predictor variables and associating them with the adopted coping strategies and the levels of psychological well-being and satisfaction with life.

## Materials and methods

### Participants and procedures

Cross-sectional study carried out with undergraduate students from 33 in-class courses at the Western Parana State University (UNIOESTE) from 5 campuses (Cascavel, Foz do Iguaçu, Francisco Beltrão, Marechal Candido Rondon and Toledo). Data collection occurred from September 14 to October 19, 2020.

The non-probabilistic convenience sampling was adopted, and the inclusion criterion was that the undergraduate must be enrolled in a classroom course at UNIOESTE and be 18 years old or older. Invitations to participate in the study were sent to undergraduate students enrolled in 2020, through the e-mails registered at the university (institutional and personal) and the dissemination of the study’s link in students’ digital media and on the university website. From the total of 8,977 enrolled undergraduates, 1,579 students responded the online research instruments and fulfilled the inclusion criteria, and 355 were excluded because did not answer the 4 scales (158 answered only the questionnaire, 19 answered only 1 scale, 123 answered only 2 scales, 55 answered only 3 scales), resulting in the final sample of 1,224 undergraduate students (13.6% of those enrolled), that responded all the questions of the applied instruments. Thus, there was no missing data to be computed.

### Measures

Data collection was performed digitally, through a link to access the online instruments on the Surveymonkey^®^ platform, containing a questionnaire prepared for the study and 4 self-applied scales.

#### Questionnaire

Questions related to sociodemographic, academic, health and pandemic effects data: sex; birth date; marital status; who you live with; do you have children; work in addition to doing internship; family income; physical activity; leisure activity; alcohol consumption; smoking; chronic disease; taking classes; had COVID-19; lives with someone who had or has COVID-19; lived with someone who died of COVID-19; did social isolation; is currently in social isolation; changes in family income, frequency of physical activity and leisure activity, or alcohol consumption during the pandemic; life aspects negatively affected by the pandemic.

#### Depression, Anxiety and Stress Scale-21 (DASS-21)

DASS-21 scale [52] validated in Brazil [53], assesses symptoms of depression, anxiety and stress, and is composed of 21 items with a 4-point Likert-type responses, referring to how the participant felt in the last week, distributed in 3 subscales with 7 questions each, for depression, anxiety and stress [53]. The scores for each subscale are the sum of the scores of the respective items multiplied by 2, classified as normal, mild, moderate, severe and extremely severe according to the following ranges: 0–9, 10–13, 14–20, 21–27, ≥28 (depression); 0–7, 8–9, 10–14, 15–19, ≥20 (anxiety); 0–14, 15–18, 19–25, 26–33, ≥34 (stress) [53].

#### Satisfaction With Life Scale (SWLS)

SWLS scale [54] validated in Brazil [55], assesses satisfaction with life and is composed of 5 items with a 7-point Likert-type responses.

#### Psychological Well-Being (PWB)

PWB scale [56] validated in Brazil for students [57], assesses psychological well-being and is composed of 36 items with a 6-point Likert-type responses, distributed in 6 subscales with 6 items each: autonomy; environmental mastery; personal growth; positive relations with others; purpose in life; self-acceptance [57].

#### BriefCOPE

BriefCOPE scale [58] validated in Brazil for students [43], measures coping reactions and is composed of 28 items with a 5-point Likert-type responses, distributed in 14 subscales with 2 items each, 8 can be associated with desirable outcomes, adaptive copings (active coping, planning, positive reframing, acceptance, humor, religion, using emotional support, using instrumental support) and 6 associated with undesirable outcomes, maladaptive copings (self-distraction, denial, venting, substance use, behavioral disengagement, self-blame) [43, 44, 59].

The scores of the SWLS and the PWB and BriefCOPE subscales were obtained by summing the scores of the respective items. Average/high scores were considered to be those equal to or greater than the central score in the range of possible scores: SWLS (5–35) ≥20; PWB subscales (6–36) ≥21; BriefCOPE (0–8) ≥4.

### Statistical analysis

Descriptive and inferential statistics were performed using the Minitab^®^ statistical software version 19.2020.1, considering a significance level of α<0.05. Microsoft Excel^®^ software was used for tabulation and coding the data. The reliability and consistency were verified by the Cronbach’s Alpha coefficient, classified as: weak (α<0.6), moderate (0.6≤α<0.7), good (0.7≤α<0.8), very good (0.8≤α<0.9) or excellent (α≥0.9) [60].

All data from scales and subscales presented non-normal distribution in the Kolmogorov-Smirnov normality test (p-value<0.010) and the Anderson-Darling normality test (p-value<0.005). The correlations between the scores on the scales were verified using the non-parametric Spearman’s correlation coefficient, and the following intervals were classified as significant: weak (0.1–0.3), moderate (0.4–0.6), strong (0.7–0.9) [61].

Univariate logistic regression was performed for the presence of symptoms of depression, anxiety and stress (binary dependent variables: normal versus symptomatic) in relation to the independent variables: 23 categorical variables (dichotomized) from the questionnaire and 21 continuous variables (SWLS, 6 PWB subscales and 14 BriefCOPE subscales). The independent variables that obtained p≤0.20 in the univariate logistic regression were included for testing in the multivariate model.

The predictor variables were determined by multivariate logistic regression (forward method, logit function, 95% confidence interval, α<0.05). The regression model’s adjustment analysis was based on the Hosmer-Lemeshow test (p>0.05 indicating that the model present good fit), and the model’s sensitivity and specificity by the analysis of the area under the ROC (Receiver Operating Characteristic) curve, considering the discrimination as: acceptable 0.7≤ROC<0.8; excellent 0.8≤ROC<0.9; outstanding ROC≥0.9 [62]. The multicollinearity was verified by VIF (Variance Inflation Factor), considering absence VIF = 1 and acceptable VIF<5 [63].

### Ethics procedures

Research Project approved by the Ethics Committee on Human Research of the Western Parana State University (judgement 4,184,430 of July 31^th^, 2020, CAAE 35209620.8.0000.0107) and with authorization from the person responsible for the study fields. The research invitation letter clarified the optional nature of participation and the guaranteed anonymity, and those interested gave digital acceptance in the Informed Consent Form before filling out the instruments.

## Results

Most of the sample was composed of female students (68.6%), between 18 and 24 years old (77.9%), single or divorced (89.4%), living accompanied (89.4%), without children (91.4%), who do not work in addition to doing internship (51.2%), have family income lower than or equal to 3 minimum wages (65.0%), perform physical activity (56.2%) and leisure activity (76.1%), alcohol consumption (54.2%), do not smoke (89.5%), were in remote classes (79.8%); do not have chronic disease (58.0%); did not have COVID-19 (86.4%); did not live with someone who had or is with COVID-19 (84.0%) or died from the disease (96.3%), made social isolation (87.2%), were not in social isolation at the time of survey participation (57.7%), and during the pandemic: kept the same or increased family income (54.4%) and the consumption of alcoholic beverages (61.6%); ceased or reduced physical activity (56.9%) and leisure activity (69.4%); had some aspect of their life negatively affected (97.3%) (Table 1). Of these aspects, the most mentioned were: studies (80.6%), mental health (66.7%) and social life (65.3%).

**Table 1:** Characterization of the socioeconomic, health, academic profile and the impact of the COVID-19 pandemic on undergraduate students, Parana, Brazil, 2020.

Variable	n (%)	Variable	n (%)
**Sex**		**Chronic disease**	
Female	840 (68.6)	No	710 (58.0)
Male	384 (31.4)	Yes	514 (42.0)
**Age (years)**		**Had COVID-19**	
18–24	953 (77.9)	No	1058 (86.4)
>24	271 (22.1)	Yes/Maybe	166 (13.6)
**Marital status**		**Lives with someone who had or has COVID-19**	
Single/Divorced	1094 (89.4)	No	1028 (84.0)
Married/Stable Union	130 (10.6)	Yes	196 (16.0)
**Who you live with**		**Lived with someone who died of COVID-19**	
Alone	130 (10.6)	No	1179 (96.3)
Accompanied	1094 (89.4)	Yes	45 (3.7)
**Children**		**Did social isolation**	
No	1119 (91.4)	No	157 (12.8)
Yes	105 (8.6)	Yes	1067 (87.2)
**Work in addition to doing internship**		**Is currently in social isolation**	
No	627 (51.2)	No	706 (57.7)
Yes	597 (48.8)	Yes	518 (42.3)
**Family income**		**Family income during the pandemic**	
≤3 minimum wages	796 (65.0)	Ceased/Reduced	558 (45.6)
>3 minimum wages	428 (35.0)	Remained the same/Increased	666 (54.4)
**Physical activity**		**Physical activity during the pandemic**	
No	536 (43.8)	Ceased/Reduced	697 (56.9)
Yes	688 (56.2)	Remained the same/Increased	527 (43.1)
**Leisure activity**		**Leisure activity during the pandemic**	
No	292 (23.9)	Ceased/Reduced	850 (69.4)
Yes	932 (76.1)	Remained the same/Increased	374 (30.6)
**Alcohol consumption**		**Alcohol consumption during the pandemic**	
No	560 (45.8)	Ceased/Reduced	470 (38.4)
Yes	664 (54.2)	Remained the same/Increased	754 (61.6)
**Smoking**		**Life aspects negatively affected by the pandemic**	
No	1095 (89.5)	No	33 (2.7)
Yes	129 (10.5)	Yes	1191 (97.3)
**Taking classes**			
No	247 (20.2)		
Yes	977 (79.8)		

The SWLS scale and PWB, BriefCOPE and DASS-21 subscales presented good or very good Cronbach’s Alpha coefficient values, with the exception of the self-blame subscale (α = 0.69) considered moderate (Table 2).

**Table 2:** Descriptive statistics and Cronbach’s alpha coefficient from the application of the DASS-21, SWLS, PWB and BriefCOPE scales in undergraduate students, Parana, Brazil, 2020.

Scale	Statistic		Frequency distribution			
(Min-Max scores; cut-off)						
**DASS-21**	**Me**	**α**	**Min**	**Max**	**Normal**	**Symptomatic**
(0–42)			**n (%)**	**n (%)**	**n (%)**	**n (%)**
Depression (>9)	12	0.92	120 (9.8)	30 (2.5)	483 (39.5)	741 (60.5)
Anxiety (>7)	8	0.89	210 (17.2)	14 (1.1)	582 (47.5)	642 (52.5)
Stress (>14)	18	0.90	57 (4.7)	35 (2.9)	520 (42.5)	704 (57.5)
	**Me**	**α**	**Min**	**Max**	**Low**	**Average/High**
			**n (%)**	**n (%)**	**n (%)**	**n (%)**
**Satisfaction with life**	20	0.85	19 (1.6)	9 (0.7)	552 (45.1)	672 (54.9)
**SWLS** (5–35; ≥20)						
**Psychological well-being**	**Me**	**α**	**Min**	**Max**	**Low**	**Average/High**
**PWB** (6–36; ≥21)			**n (%)**	**n (%)**	**n (%)**	**n (%)**
Autonomy	23	0.72	4 (0.3)	8 (0.7)	403 (32.9)	821 (67.1)
Environmental mastery	21	0.70	3 (0.2)	3 (0.2)	556 (45.4)	668 (54.6)
Personal growth	33	0.73	0 (0.0)	193 (15.8)	19 (1.6)	1205 (98.4)
Positive relations with others	23	0.74	2 (0.2)	22 (1.8)	438 (35.8)	786 (64.2)
Purpose in life	27	0.82	2 (0.2)	47 (3.8)	242 (19.8)	982 (80.2)
Self-acceptance	25	0.86	11 (0.9)	37 (3.0)	380 (31.0)	844 (69.0)
**Adaptive coping**	**Me**	**α**	**Min**	**Max**	**Low**	**Average/High**
**BriefCOPE** (0–8; ≥4)			**n (%)**	**n (%)**	**n (%)**	**n (%)**
Active coping	5	0.73	8 (0.7)	97 (7.9)	213 (17.4)	1011 (82.6)
Planning	6	0.71	5 (0.4)	198 (16.2)	121 (9.9)	1103 (90.1)
Positive reframing	4	0.86	45 (3.7)	122 (10.0)	373 (30.5)	851 (69.5)
Acceptance	5	0.70	13 (1.1)	69 (5.6)	299 (24.4)	925 (75.6)
Humor	2	0.88	200 (16.3)	71 (5.8)	747 (61.0)	477 (39.0)
Religion	3	0.90	213 (17.4)	196 (16.0)	618 (50.5)	606 (49.5)
Using emotional support	4	0.88	90 (7.4)	189 (15.4)	475 (38.8)	749 (61.2)
Using instrumental support	4	0.84	53 (4.3)	149 (12.2)	413 (33.7)	811 (66.3)
**Maladaptive coping**	**Me**	**α**	**Min**	**Max**	**Low**	**Average/High**
**BriefCOPE** (0–8; ≥4)			**n (%)**	**n (%)**	**n (%)**	**n (%)**
Self-distraction	4	0.78	57 (4.7)	136 (11.1)	392 (32.0)	832 (68.0)
Denial	2	0.73	301 (24.6)	21 (1.7)	910 (74.3)	314 (25.7)
Venting	4	0.92	62 (5.1)	188 (15.4)	441 (36.0)	783 (64.0)
Substance use	0	0.94	730 (59.6)	36 (2.9)	1034 (84.5)	190 (15.5)
Behavioral disengagement	2	0.90	344 (28.1)	40 (3.3)	923 (75.4)	301 (24.6)
Self-blame	6	0.69	4 (0.3)	259 (21.2)	209 (17.1)	1015 (82.9)

Min, minimum score; Max, maximum score; Me, Median; α, Cronbach’s alpha.

For the psychological well-being (PWB) subscales, the median of the scores obtained by the surveyed undergraduates in all subscales was equal to or higher than the average score (21), indicating that in most subscales there was a higher percentage of students with high scores. Higher frequencies of average/high scores were observed in personal growth (98.4%) and purpose in life (80.2%), and the lowest frequency of average/high scores were observed in environmental mastery (54.6%). About half of undergraduates (54.9%) had average/high scores for satisfaction with life (Table 2).

Among maladaptive copings, the most used was self-blame, with a high percentage of average/high scores (82.9%) and maximum scores (21.2%). Substance use was the least used, with a high percentage of low scores (84.5%) and minimum scores (59.6%). In relation to adaptive coping, planning stood out due to the high percentage of average/high scores (90.1%). The adaptive copings humor and religion stood out for the low percentages of average/high scores, 39.0% and 49.5%, respectively (Table 2).

The prevalence of depression, anxiety and stress at symptomatic levels (mild to extremely severe) was 60.5%, 52.5% and 57.5%, respectively (Table 2).

In the surveyed undergraduates, the percentages of depression, anxiety and stress symptoms at severe and extremely severe levels were 29.8%, 30.9% and 28.2%, respectively (Table 3). In addition, 26.1% of the undergraduates showed a combination of depression, anxiety and stress symptoms (n = 319).

**Table 3:** Frequency distribution of depression, anxiety and stress levels in undergraduate students, Parana, Brazil, 2020.

	Frequency distribution					
	Normal	Mild	Moderate	Severe	Extremely Severe	Total
	n (%)	n (%)	n (%)	n (%)	n (%)	n (%)
Depression	483 (39.5)	146 (11.9)	231 (18.9)	116 (9.5)	248 (20.3)	1224 (100.0)
Anxiety	582 (47.5)	75 (6.1)	189 (15.4)	92 (7.5)	286 (23.4)	1224 (100.0)
Stress	520 (42.5)	153 (12.5)	206 (16.8)	183 (15.0)	162 (13.2)	1224 (100.0)

Depression, anxiety, and stress symptoms showed significant correlations in common: negative (weak or moderate) with satisfaction with life, all subscales of psychological well-being, and 3 adaptive copings (active coping, planning, positive reframing); positive (weak or moderate) with 5 maladaptive copings (self-distraction, denial, substance use, behavioral disengagement, self-blame). In addition to the common correlations, weak positive correlations of stress and anxiety were found with 1 maladaptive coping (venting) and weak negative correlations: of depression with 3 adaptive copings (religion, using emotional support, using instrumental support); of stress with 2 adaptive copings (acceptance, religion). Satisfaction with life showed significant positive correlations (weak, moderate or strong) with all subscales of psychological well-being. Satisfaction with life and psychological well-being scales showed significant correlations (weak or moderate): positive with 4 adaptive copings (active coping, planning, positive reframing, acceptance); negative with 3 maladaptive copings (substance use, behavioral disengagement, self-blame) (Table 4).

**Table 4:** Correlations among depression, anxiety, stress, satisfaction with life, psychological well-being and coping strategies in undergraduate students, Parana, Brazil, 2020.

Variables	1	2	3	4	5	6	7	8	9	10	11	12	13	14	15	16	17	18	19	20	21	22	23	24
1. Depression	1																							
2. Anxiety	.699^§^	1																						
3. Stress	.742^§^	.796^§^	1																					
4. Satisfaction with life	-.541^§^	-.310^§^	-.368^§^	1																				
**Psychological well-being**																								
5. Autonomy	-.301^§^	-.259^§^	-.274^§^	.232^§^	1																			
6. Environmental mastery	-.503^§^	-.381^§^	-.409^§^	.493^§^	.362^§^	1																		
7. Personal growth	-.336^§^	-.187^§^	-.205^§^	.408^§^	.312^§^	.422^§^	1																	
8. Positive relations with others	-.470^§^	-.324^§^	-.323^§^	.410^§^	.270^§^	.313^§^	.369^§^	1																
9. Purpose in life	-.592^§^	-.344^§^	-.375^§^	.653^§^	.333^§^	.642^§^	.614^§^	.456^§^	1															
10. Self-acceptance	-.687^§^	-.438^§^	-.488^§^	.723^§^	.425^§^	.616^§^	.524^§^	.508^§^	.807^§^	1														
**Adaptive coping**																								
11. Active coping	-.322^§^	-.198^§^	-.234^§^	.415^§^	.273^§^	.443^§^	.432^§^	.304^§^	.498^§^	.471^§^	1													
12. Planning	-.270^§^	-.162^§^	-.172^§^	.318^§^	.273^§^	.372^§^	.420^§^	.250^§^	.441^§^	.394^§^	.626^§^	1												
13. Positive reframing	-.343^§^	-.197^§^	-.259^§^	.388^§^	.247^§^	.362^§^	.411^§^	.349^§^	.462^§^	.462^§^	.444^§^	.439^§^	1											
14. Acceptance	-.099*	-.072*	-.104^§^	.202^§^	.100^§^	.128^§^	.249^§^	.171^§^	.198^§^	.181^§^	.265^§^	.270^§^	.389^§^	1										
15. Humor	-.074*	-.020	-.039	.082*	.086*	.023	.063*	.155^§^	.021	.079*	.099^§^	.095*	.238^§^	.235^§^	1									
16. Religion	-.256^§^	-.091*	-.119^§^	.263^§^	.026	.214^§^	.228^§^	.160^§^	.345^§^	.279^§^	.181^§^	.156^§^	.304^§^	.091*	-.045	1								
17. Using emotional support	-.115^§^	.042	.043	.247^§^	-.090*	.065*	.205^§^	.252^§^	.218^§^	.148^§^	.170^§^	.126^§^	.171^§^	.148^§^	.029	.217^§^	1							
18. Using instrumental support	-.114^§^	.000	.000	.215^§^	-.064*	.098*	.227^§^	.280^§^	.225^§^	.158^§^	.266^§^	.257^§^	.227^§^	.167^§^	.109^§^	.115^§^	.594^§^	1						
**Maladaptive coping**																								
19. Self-distraction	.224^§^	.210^§^	.238^§^	-.144^§^	-.157^§^	-.188^§^	-.022	-.059*	-.163^§^	-.222^§^	-.051	-.001	-.014	.145^§^	.146^§^	-.042	.130^§^	.114^§^	1					
20. Denial	.225^§^	.281^§^	.265^§^	-.098*	-.210^§^	-.137^§^	-.068*	-.142^§^	-.071*	-.166^§^	-.092*	-.080*	.010	-.021	.008	.099^§^	.134^§^	.050	.227^§^	1				
21. Venting	.085*	.192^§^	.247^§^	.041	-.081*	-.066*	.081*	.052	.036	-.030	.022	.034	.074*	.090*	-.000	.142^§^	.408^§^	.266^§^	.182^§^	.258^§^	1			
22. Substance use	.326^§^	.328^§^	.321^§^	-.253^§^	-.116^§^	-.261^§^	-.128^§^	-.107^§^	-.253^§^	-.277^§^	-.167^§^	-.158^§^	-.144^§^	-.005	.097*	-.166^§^	.011	.026	.183^§^	.191^§^	.110^§^	1		
23. Behavioral disengagement	.460^§^	.300^§^	.325^§^	-.416^§^	-.310^§^	-.460^§^	-.333^§^	-.273^§^	-.564^§^	-.553^§^	-.432^§^	-.359^§^	-.263^§^	-.032	.048	-.141^§^	-.045	-.083*	.256^§^	.251^§^	.111^§^	.295^§^	1	
24. Self-blame	.472^§^	.406^§^	.456^§^	-.343^§^	-.275^§^	-.346^§^	-.163^§^	-.268^§^	-.374^§^	-.492^§^	-.122^§^	-.063*	-.197^§^	.060*	.010	-.135^§^	.024	.060*	.271^§^	.228^§^	.175^§^	.249^§^	.337^§^	1

*p<0.05

^§^p<0.001.

Univariate logistic regression was performed for the presence of symptoms of depression, anxiety and stress in relation to the independent variables (23 categorical and 21 continuous), and variables with p<0.20 were included in the multivariate analysis: 38 for depression; 34 for stress and anxiety (Tables 5–7).

**Table 5:** Univariate and multivariate logistic regression of independent variables in relation to symptoms of depression in undergraduate students, Parana, Brazil, 2020.

	Univariate regression		Multivariate regression^§^		
Variables	OR (95%IC)	p	OR (95%IC)	p	VIF
Sex^1^	1.665 (1.303–2.127)	<0.001	2.287 (1.620–3.229)	<0.001	1.13
Age (years)^2^	1.396 (1.063–1.833)	0.017	1.751 (1.185–2.585)	0.005	1.14
Children^3^	0.638 (0.427–0.953)	0.028			
Familiar income^4^	1.253 (0.987–1.592)	0.064*			
Taking classes^3^	1.222 (0.921–1.621)	0.165*			
Work in addition to doing internship^3^	0.686 (0.545–0.864)	0.001	0.677 (0.493–0.929)	0.016	1.09
Physical activity^5^	1.648 (1.303–2.084)	<0.001			
Leisure activity^5^	2.203 (1.646–2.950)	<0.001			
Smoking^3^	1.453 (0.984–2.145)	0.060*			
Chronic disease^3^	2.176 (1.710–2.769)	<0.001	1.533 (1.115–2.108)	0.009	1.03
Lived with someone who died of COVID-19^3^	1.829 (0.935–3.578)	0.078*			
Did social isolation^3^	1.567 (1.119–2.194)	0.009			
Is currently in social isolation^3^	1.474 (1.165–1.864)	0.001			
Familiar income during the pandemic^6^	2.241 (1.767–2.842)	<0.001	1.465 (1.072–2.001)	0.016	1.02
Physical activity during the pandemic^6^	1.652 (1.310–2.083)	<0.001			
Leisure activity during the pandemic^6^	1.523 (1.190–1.949)	0.001			
Life aspects negatively affected by pandemic^3^	2.419 (1.192–4.910)	0.014			
Satisfaction with life	0.849 (0.830–0.868)	<0.001	0.949 (0.919–0.980)	0.002	1.63
**Psychological well-being**					
Autonomy	0.914 (0.895–0.933)	<0.001			
Environmental mastery	0.849 (0.828–0.870)	<0.001			
Personal growth	0.854 (0.823–0.885)	<0.001			
Positive relations with others	0.859 (0.840–0.879)	<0.001	0.925 (0.899–0.952)	<0.001	1.11
Purpose in life	0.813 (0.791–0.836)	<0.001			
Self-acceptance	0.792 (0.771–0.814)	<0.001	0.875 (0.841–0.911)	<0.001	1.91
**Adaptive coping**					
Active coping	0.727 (0.674–0.785)	<0.001	1.164 (1.042–1.302)	0.007	1.36
Planning	0.748 (0.693–0.808)	<0.001			
Positive reframing	0.748 (0.705–0.795)	<0.001			
Acceptance	0.906 (0.848–0.968)	0.003			
Humor	0.940 (0.893–0.990)	0.019			
Religion	0.854 (0.818–0.891)	<0.001	0.912 (0.860–0.969)	0.003	1.15
Using emotional support	0.931 (0.888–0.976)	0.003			
Using instrumental support	0.908 (0.863–0.957)	<0.001			
**Maladaptive coping**					
Self-distraction	1.211 (1.146–1.280)	<0.001			
Denial	1.247 (1.169–1.330)	<0.001	1.100 (1.005–1.203)	0.038	1.13
Venting	1.068 (1.016–1.122)	0.009			
Substance use	1.340 (1.244–1.444)	<0.001	1.121 (1.020–1.233)	0.018	1.13
Behavioral disengagement	1.601 (1.482–1.730)	<0.001	1.167 (1.049–1.299)	0.005	1.37
Self-blame	1.552 (1.449–1.662)	<0.001	1.145 (1.047–1.252)	0.003	1.17

Odd Ratio (OR) references

^1^male

^2^>24

^3^no

^4^ >3 minimum salaries

^5^yes

^6^remained the same/increased.

^§^R^2^ 37.2%; R^2^ adj 36.4%; ROC 0.882; Hosmer-Lemeshow 0.090.

*p<0.20.

**Table 6:** Univariate and multivariate logistic regression of independent variables in relation to symptoms of anxiety in undergraduate students, Parana, Brazil, 2020.

	Univariate regression		Multivariate regression^§^		
Variables	OR (95%IC)	p	OR (95%IC)	p	VIF
Sex^1^	2.171 (1.697–2.778)	<0.001	2.132 (1.581–2.875)	<0.001	1.08
Age (years)^2^	1.359 (1.037–1.781)	0.026	1.432 (1.031–1.988)	0.032	1.03
Children^3^	0.684 (0.457–1.024)	0.065*			
Familiar income^4^	1.324 (1.046–1.676)	0.019			
Taking classes^3^	1.239 (0.937–1.639)	0.133*			
Physical activity^5^	1.573 (1.253–1.976)	<0.001			
Leisure activity^5^	1.285 (0.986–1.675)	0.063			
Smoking^3^	1.796 (1.227–2.629)	0.003			
Chronic disease^3^	2.640 (2.086–3.341)	<0.001	1.921 (1.458–2.532)	<0.001	1.02
Had COVID-19^3^	1.442 (1.033–2.013)	0.031			
Lived with someone who died of COVID-19^3^	2.296 (1.193–4.419)	0.013			
Did social isolation^3^	1.436 (1.025–2.011)	0.035			
Familiar income during the pandemic^6^	1.958 (1.558–2.462)	<0.001	1.462 (1.115–1.918)	0.006	1.02
Physical activity during the pandemic^6^	1.368 (1.090–1.716)	0.007			
Leisure activity during the pandemic^6^	1.453 (1.138–1.854)	0.003			
Life aspects negatively affected by pandemic^3^	1.965 (0.958–4.031)	0.065*			
**Satisfaction with life**	0.926 (0.910–0.942)	<0.001			
**Psychological well-being**					
Autonomy	0.919 (0.900–0.938)	<0.001			
Environmental mastery	0.887 (0.868–0.906)	<0.001	0.954 (0.927–0.982)	0.001	1.42
Personal growth	0.922 (0.894–0.950)	<0.001			
Positive relations with others	0.904 (0.886–0.922)	<0.001	0.944 (0.920–0.968)	<0.001	1.25
Purpose in life	0.905 (0.887–0.923)	<0.001			
Self-acceptance	0.891 (0.875–0.908)	<0.001	0.971 (0.945–0.998)	0.034	1.83
**Adaptive coping**					
Active coping	0.821 (0.766–0.880)	<0.001			
Planning	0.854 (0.797–0.914)	<0.001			
Positive reframing	0.838 (0.793–0.885)	<0.001			
Acceptance	0.907 (0.850–0.967)	0.003			
Religion	0.970 (0.931–1.010)	0.142*			
**Maladaptive coping**					
Self-distraction	1.202 (1.138–1.269)	<0.001			
Denial	1.333 (1.249–1.422)	<0.001	1.156 (1.071–1.249)	<0.001	1.08
Venting	1.160 (1.104–1.220)	<0.001	1.070 (1.004–1.140)	0.036	1.16
Substance use	1.360 (1.269–1.459)	<0.001	1.202 (1.110–1.302)	<0.001	1.09
Behavioral disengagement	1.333 (1.254–1.418)	<0.001			
Self-blame	1.498 (1.402–1.601)	<0.001	1.221 (1.126–1.323)	<0.001	1.22

Odd Ratio (OR) references

^1^male

^2^>24

^3^no

^4^>3 minimum salaries

^5^yes

^6^remained the same/increased.

^§^R^2^ 24.1%; R^2^ adj 23.4%; ROC 0.814; Hosmer-Lemeshow 0.624.

*p<0.20.

**Table 7:** Univariate and multivariate logistic regression of independent variables in relation to symptoms of stress in undergraduate students, Parana, Brazil, 2020.

	Univariate regression		Multivariate regression^§^		
Variables	OR (95%IC)	p	OR (95%IC)	p	VIF
Sex^1^	2.063 (1.615–2.636)	<0.001	1.872 (1.380–2.540)	<0.001	1.10
Age (years)^2^	1.674 (1.276–2.197)	<0.001	1.990 (1.424–2.781)	<0.001	1.05
Children^3^	0.481 (0.320–0.722)	<0.001			
Work in addition to doing internship^3^	0.814 (0.649–1.021)	0.075*			
Physical activity^4^	1.364 (1.083–1.716)	0.008			
Leisure activity^4^	1.457 (1.110–1.913)	0.007			
Smoking^3^	1.544 (1.050–2.269)	0.027			
Alcohol consumption^3^	1.193 (0.950–1.497)	0.129*			
Chronic disease^3^	2.344 (1.847–2.974)	<0.001	1.689 (1.272–2.242)	<0.001	1.02
Had COVID-19^3^	1.280 (0.914–1.794)	0.151*			
Did social isolation^3^	1.438 (1.028–2.011)	0.034			
Familiar income during the pandemic^5^	1.799 (1.428–2.267)	<0.001			
Physical activity during the pandemic^5^	1.389 (1.105–1.746)	0.005			
Leisure activity during the pandemic^5^	1.577 (1.234–2.015)	<0.001	1.370 (1.018–1.844)	0.038	1.03
Life aspects negatively affected by pandemic^3^	3.212 (1.515–6.808)	0.002			
**Satisfaction with life**	0.907 (0.890–0.924)	<0.001			
**Psychological well-being**					
Autonomy	0.916 (0.898–0.936)	<0.001			
Environmental mastery	0.874 (0.855–0.894)	<0.001	0.951 (0.922–0.980)	0.001	1.43
Personal growth	0.927 (0.898–0.956)	<0.001	1.050 (1.006–1.096)	0.026	1.48
Positive relations with others	0.917 (0.899–0.935)	<0.001	0.967 (0.942–0.992)	0.010	1.30
Purpose in life	0.892 (0.873–0.911)	<0.001			
Self-acceptance	0.872 (0.854–0.889)	<0.001	0.920 (0.893–0.948)	<0.001	2.01
**Adaptive coping**					
Active coping	0.782 (0.728–0.840)	<0.001			
Planning	0.852 (0.795–0.914)	<0.001			
Positive reframing	0.799 (0.755–0.846)	<0.001			
Acceptance	0.918 (0.860–0.980)	0.010			
Religion	0.925 (0.887–0.964)	<0.001			
Using emotional support	1.031 (0.985–1.080)	0.193*			
**Maladaptive coping**					
Self-distraction	1.242 (1.174–1.313)	<0.001			
Denial	1.279 (1.199–1.364)	<0.001			
Venting	1.189 (1.131–1.251)	<0.001	1.133 (1.063–1.207)	<0.001	1.11
Substance use	1.391 (1.290–1.500)	<0.001	1.238 (1.135–1.350)	<0.001	1.07
Behavioral disengagement	1.403 (1.313–1.500)	<0.001			
Self-blame	1.529 (1.429–1.635)	<0.001	1.215 (1.121–1.318)	<0.001	1.19

Odd Ratio (OR) references

^1^male

^2^>24

^3^no

^4^yes

^5^remained the same/increased.

^§^R^2^ 25.3%; R^2^ adj 24.7%; ROC 0.822; Hosmer-Lemeshow 0.340.

*p<0.20.

The multivariate model for depression symptoms in undergraduates (Adjusted R^2^ = 36.4%; ROC = 0.882 [excellent]; Hosmer-Lemeshow = 0.090 [good]; VIF between 1.02 to 1.91 [acceptable]) found 14 predictor variables: female; age 18–24 years old; not having work in addition to doing internship; having chronic illness; family income at pandemic has ceased or reduced; lower scores of satisfaction with life; lower scores of positive relations with others and self-acceptance on the psychological well-being scale; lower scores for adaptive coping religion and higher scores for adaptive coping active coping; higher scores of maladaptive copings denial, substance use, behavioral disengagement and self-blame (Table 5).

The multivariate model for anxiety symptoms in undergraduates (Adjusted R^2^ = 23.4%; ROC = 0.814 [excellent]; Hosmer-Lemeshow = 0.624 [good]; VIF between 1.02 to 1.83 [acceptable]) found 11 predictor variables: female; age 18–24 years old; having chronic illness; family income at pandemic has ceased or reduced; lower scores of environmental mastery, positive relations with others and self-acceptance; higher scores of maladaptive copings denial, venting, substance use and self-blame (Table 6).

The multivariate model for stress symptoms in undergraduates (Adjusted R^2^ = 24.7%; ROC = 0.822 [excellent]; Hosmer-Lemeshow = 0.340 [good]; VIF between 1.02 to 2.01 [acceptable]) found 11 predictor variables: female; age 18–24 years old; having chronic illness; leisure activity ceased or reduced in the pandemic; higher scores of personal growth and lower scores of environmental mastery, positive relations with others and self-acceptance, higher scores of maladaptive copings venting, substance use and self-blame (Table 7).

## Discussion

### Prevalence of depression, anxiety and stress symptoms

The results indicate that the majority of undergraduates surveyed presented mild to severe symptoms of depression (60.5%), anxiety (52.5%) and stress (57.5%). Compared with other studies during the COVID-19 pandemic, using the same instrument, the percentages of the present study were higher than those found in the Brazilian adult population for depression (21.5%, 46.4% and 54.5%), anxiety (19.4%, 39.7% and 41.1%) and stress (21.5%, 42.2% and 45.7%) [7, 9, 10], and in the population of 8 countries (China, Spain, Italy, Iran, USA, Turkey, Nepal, Denmark) for depression (14.6% to 48.3%) and anxiety (6.3% to 50.9%) [5].

Regarding stress, the result of the surveyed students was higher than those of undergraduates from Bangladesh [28], Egypt [26], Ethiopia [24, 30], Lebanon [25] and Turkey [29], whose percentages ranged from 12.7% to 47.8%. For anxiety, the percentage was lower than those in Egypt (53.6%) and higher than the others (27.7% to 48.6%). For depression, the percentage was lower than those of Egypt and Turkey (70.5% and 64.6%) and higher than others (21.2% to 46.9%). Another study in undergraduates from Bangladesh [27] found higher values than all mentioned studies for depression (76.1%), anxiety (71.5%) and stress (70.1%).

The higher percentages of depression, anxiety and stress symptoms in surveyed undergraduates in relation to the Brazilian population may be related to the fact that the data collection of the present research occurred in a more advanced period of the pandemic in 2020 than the data collection of the cited studies [7, 9, 10].

Most undergraduates reported having had at least one life aspect negatively affected by the COVID-19 pandemic (97.3%), notably the studies (80.6%). During the period of data collection, most respondents had restarted academic activities remotely (79.8%) about 1 or 2 months ago (August 17, 2020), after the interruption of the school year. Thus, the high prevalence of depression, anxiety and stress symptoms also suggests the additional psychological impact of the pandemic on students due to repercussions on education, such as adaptation to remote classes, as assessed in other studies [10, 15, 16, 25].

### Prevalence of satisfaction with life and psychological well-being

In this study, undergraduates obtained average scores for satisfaction with life (20.1) lower than that found in technology students in Poland during the pandemic (22.0) [48] and in medical students from the state of Pernambuco in Brazil, before the pandemic (22.3) [40].

Most of the surveyed undergraduates presented average/high scores in the 6 dimensions of the psychological well-being scale (54.6% to 98.4%) and the lowest frequency of average/high scores were observed in environmental mastery dimension (54.6%). A longitudinal study with Spanish university students, which divided the study participations into 3 groups (ordinary week, pre-confinement and confinement in the pandemic), observed a decrease in the environmental mastery between the ordinary week and the pre-confinement [64]. In the context of the COVID-19 pandemic, with restrictions imposed by public health protocols, the low scores for environmental mastery in the surveyed undergraduates may be influenced by the difficulties in choosing and creating appropriate contexts to meet their personal needs as a result of isolation and social distancing.

A study suggests that subjective well-being, involving satisfaction with life and negative and positive affects, tends to be strongly influenced by emotional experiences, which vary over time and context, and psychological well-being tends to be based on more stable abilities [65]. Thus, in the present research, the lower scores for satisfaction with life suggest that these levels are being more affected by the pandemic crisis compared to levels of psychological well-being among undergraduates surveyed.

### Use of coping strategies

The frequency of average/high scores for the adaptive coping active coping (82.6%) and planning (90.1%) stands out in the sample, indicating that most of the surveyed undergraduate students tend to think about the best way to deal with the problem when facing a stressful situation and try to take actions to eliminate, minimize or circumvent it.

The surveyed undergraduates had a low frequency of average/high scores (39.0%) regarding the adaptive coping humor and it had no significant correlation with symptoms of depression, anxiety and stress, diverging from another study in the pandemic with university students in France that found negative correlations of humor with anxiety and depression [66].

### Correlations between the studied dimensions

The results of the correlation analysis showed that the higher levels of satisfaction with life and the 6 dimensions of psychological well-being, in addition to higher scores of 3 adaptive coping (active coping, planning, positive reframing) may act as protective factors against symptoms of depression, anxiety and stress, as well as higher scores of 5 maladaptive copings (self-distraction, denial, substance use, behavioral disengagement, self-blame) may be risk factors for such symptoms. These results corroborate the data of other studies with students during the COVID-19 pandemic: in China, it was found that adaptive coping may, along with resilience and social support, mitigate the association between pandemic stressful events and stress symptoms [49]; in France, depression symptoms were positively correlated with 4 maladaptive copings (substance use, denial, behavioral disengagement, self-blame) and negatively correlated with the 3 adaptive copings (active coping, planning and positive reframing) [66]; in Poland, in technology students, anxiety was positively correlated with emotion-oriented copings (self-blame, irritation, getting tense, worrying, fantasizing) and negatively correlated with satisfaction with life [48].

Studies prior to the pandemic, using the same instruments, found: in Australian university students, negative correlations between all dimensions of psychological well-being and depression [39]; in the Portuguese population, positive correlations of all dimensions of psychological well-being with the adaptive coping active coping and negative correlations with maladaptive copings self-distraction, denial, substance use and behavioral disengagement [45].

### Predictors of depression, anxiety and stress symptoms

In the present study, being female, aged between 18 and 24 years old, having a chronic disease, presenting lower scores in the dimensions of psychological well-being (positive relations with others and self-acceptance), and higher scores of maladaptive copings substance use and self-blame were common predictors of depression, anxiety and stress symptoms in undergraduate students during the COVID-19 pandemic.

Studies in the Brazilian population during the pandemic suggest that being a woman, younger and having a chronic disease were among the predictors of severe anxiety symptoms [10] and were associated with depression and anxiety symptoms [36]. Being female and younger were among the predictors for severe symptoms of depression and stress [10] and were associated with greater severity of symptoms of depression, anxiety and stress [8].

A review study of the population mental health during the pandemic, including 8 countries (China, Spain, Italy, Iran, USA, Turkey, Nepal and Denmark) suggested that being a woman, up to 40 years old and having chronic or psychiatric illness were among the risk factors associated with stress, in addition to other variables such as being a student, being unemployed and frequently exposed to social media and news about COVID-19 [5]. In the Italian population, a study showed that being a woman was among the predictors for depression, anxiety and stress; being young was among the predictors of stress; and having a history of medical problems was among the predictors of depression and anxiety [67]. In the Chinese population, a study found that being a woman, a student, having specific physical symptoms, and evaluating oneself as being in poor health were associated with high levels of depression, anxiety and stress [68].

In Egyptian students, female gender and chronic illness, in addition to having a family member or acquaintance infected with COVID-19 and lack of psychological support, increased the risk of depression, anxiety and stress symptoms [26]. In Ethiopian students, female gender and history of medical illness, in addition to being at home and having little social support, increased the risk of depression symptoms [24].

A review study before the pandemic identified that female gender was among the sociodemographic characteristics most associated with psychological distress in undergraduates [12]. During the pandemic, this variable was associated with symptoms of depression, anxiety and stress in several studies in students [24, 26, 27, 29] and in populations in Brazil [7–10] and other countries [5, 67, 68]. Such a condition points to the need for further studies aiming to identify possible common causes of female susceptibility to stress during a pandemic crisis, regardless of age and culture.

In addition to the vulnerabilities to stress already identified in undergraduates in periods prior the pandemic [12] and the stressors that emerged in the pandemic crisis [16], younger students (18 to 24 years old) tend to suffer greater impact with social distancing, as it hinders the natural movement of this population to seek greater closeness with their peers [15]. Thus, it is possible to think that adults tend to have developed adaptation and coping mechanisms when facing stressful situations, being able to better preserve their own mental health.

The pandemic crisis has impacted the chronically ill population´s mental health [5, 9, 10, 19, 24, 26, 36, 67] and can be explained by the uncertainties involving the risks of worsening and mortality by COVID-19 resulting from pre-existing diseases.

Lower scores for self-acceptance (dissatisfaction with yourself, disappointment with past events, concern with certain personal qualities and desire to be different from what you are [69]) and positive relations with others (few stable and trusting relationships, difficulty in being warm, open and concerned about others [69]) are opposite conditions to some protective factors for psychological distress in students found in the scientific literature, such as self-esteem, talking to friends, communication skills and social engagement [12]. In a pandemic context, restrictions on face-to-face contacts may increase lonely moments, especially in those who tend to isolation and have few satisfying empathic interactions, which can intensify stress and associated disorders in those with difficulties in feeling good about themselves.

The maladaptive coping substance use was a common predictor of depression, anxiety and stress. A study in university students in Ethiopia also found the use of substances, such as tobacco and alcohol, in the last 3 months during the pandemic, among the predictors of anxiety [30]. In the present study, there was a low percentage of substance use (15.5%), diverging from what was found in a study carried out in the Brazilian population, in which 40.8% reported having increased the use of drugs, medications, tobacco or food during the pandemic [9].

Most of the surveyed students (82.9%) showed average/high use of the coping self-blame (criticizing oneself for responsibility in the situation [43]) and this coping was a predictor of depression, anxiety and stress symptoms. Self-blame often occurs after a stressful event that can generate negative outcomes, to which the responsibility, causality and/or intentionality is attributed to oneself, originated frequently from a distorted cognition [70]. It is possible to think that the pandemic crisis may have intensified the tendency to blame oneself for adverse events in those predisposed to use such maladaptive coping. A study in United States undergraduates, before the pandemic, found that a high level of so-called proactive coping strategies (stressors perceived as potential challenges rather than threats) were predictors of low levels of self-blame and stress, and the authors suggest the implementation of actions to assist undergraduates in using proactive coping to deal with stressful situations [70].

In the present study, stress and anxiety symptoms had as a common predictor lower scores of environmental mastery, which can be understood as difficulty in managing daily issues and inability to see opportunities to change or improve the context [56, 71]. It can be thought that the pandemic crisis and the resulting restrictive measures tend to make actions impossible and restrict opportunities.

Another common predictor of stress and anxiety symptoms was higher scores of the coping venting, which can be understood as the exposure or the communication of emotional stress to others [59], being consistent with the evasion characteristic attributed to this coping, as keeping the focus on annoyance distracts oneself from the efforts required to eliminate or reduce stress [59].

Symptoms of depression and anxiety had as predictors cessation or reduction of family income during the pandemic. The result is consistent with other studies in the Brazilian population that found the decrease in family income among the predictors of severe symptoms of depression [10] and associated with higher percentages of depression and anxiety symptoms [36]. A review study of previous pandemics points out that financial losses are problems during and after the quarantine period and are risk factors for psychological disorders symptoms [2], which warns that the pandemic economic effects tend to last and be a risk factor for psychological disorders for students and the population.

Another predictor of depression and anxiety was higher scores of coping denial (refusal to believe that the stressor exists or trying to act as if the stressor is not real [59]), which reinforces the argument that denial can create additional problems because it allows the stressful event to become more serious, making it more difficult to cope with [59], especially in a pandemic context, in which effective actions are necessary for COVID-19 prevention, as well as to overcome the possible educational, social and economic difficulties resulting from the pandemic crisis.

In addition, stress symptoms had as predictors the condition of having paralyzed or reduced leisure activities during the pandemic and higher scores of personal growth (sense of continuous development with the personal potentials realization) [56, 71].

Reduction of leisure was among the predictors of severe symptoms of depression, anxiety and stress in a study in the Brazilian population [10]. In Bangladeshi students, limited or lack of recreational activities were associated with stress, anxiety and depression [28]. These results suggest the importance of relaxation produced by leisure in preserving mental health. A pre-pandemic study in Chinese students suggested that effectively reducing academic stress and engaging in leisure activities are both important in promoting and improving emotional well-being [72].

In relation to the higher personal growth scores, it is possible to think that undergraduates who value continuous growth may have had an increase in stress levels due to the impossibilities of the pandemic moment, especially in relation to studies and professional training.

The predictors of depressive symptoms were not working in addition to doing internship, lower scores of satisfaction with life and the coping religion and higher scores of active coping and behavioral disengagement.

In this study, 49.6% of the surveyed undergraduates who do not work reported having ceased or reduced family income during the pandemic and of these, 90.7% do not live alone, suggesting loss of employment or need for help in the family, which may explain the favoring of depressive symptoms, because unemployment was found among the predictors of depression in pandemic studies in several countries [5].

The presence of higher scores of active coping among the predictors of depression can be analyzed based on the questioning of Carver, Scheier and Weintraub (1989) about what would happen to a person who prefers to use active coping when the situation requires restriction or moderation [59]. The results suggest that the restrictions imposed by the pandemic crisis, which make it impossible or difficult to take actions to remove, circumvent or lessen the effects of the stressor, could cause depressive symptoms, such as loss of incentive and low perceived probability of achieving meaningful goals [52, 53], in those who tend to use active coping as a stress coping strategy.

Lower scores of satisfaction with life (poorer personal judgment about how satisfied someone is with your current life, regardless of objective assessments about quality of life) [54], higher scores for the coping behavioral disengagement (giving up goals related to the stressor element) [43, 59] and lower scores of the coping religion (low or no participation in religious activities) [43, 59] could favor the emergence of the following depressive symptoms: devaluation of life; lack of interest and involvement; hopelessness [52, 53]. Not practicing religion was one of the predictors of severe depression symptoms in the Brazilian population [10].

The results of the present study showed that satisfaction with life, dimensions of psychological well-being and coping strategies were associated and/or predictors of symptoms of depression, anxiety and/or stress during the pandemic crisis.

In general, the levels of personal growth and purpose in life tend to be higher in younger people, tending to decrease with age when there are no opportunities to engage in meaningful activities, and the maintenance of high levels throughout life have been associated with health benefits [37]. The majority of the surveyed undergraduates, composed mostly of young people aged 18 to 24 years old, presented high personal growth scores (98.4%) and purpose in life (80.2%), which raises questions about how to help them conserve such levels.

The psychological well-being and satisfaction with life, even though related to the personality and considered relatively stables, despite short-term fluctuations resulting from transitory events, the personality traits may change after specific experiences and interventions [73]. Similarly, coping strategies can be learned responses, initiated voluntarily and, with repetition, become involuntary [43]. Therefore, actions may be proposed to help undergraduates to obtain higher scores of adaptive coping and dimensions of psychological well-being.

### Study limitations

The convenience sampling does not allow generalizing the results to the totality of Brazilian university students or the researched university. However, since the sample includes students from 5 campuses (in different municipalities) located in the western region of southern Brazil and from 33 undergraduate courses at the university, it can be considered broad and representative.

A cross-sectional study does not allow causality inferences and to evaluate the long-term impacts of the pandemic crisis, however, it presents relevant evidence that can foster future longitudinal studies considering the relations of the levels of depression, anxiety and stress symptoms with life satisfaction, psychological well-being and coping strategies.

The use of self-report measures is also a limitation of the present study. However, the fact that the four scales applied in this research were international and the versions used had been validated in Brazil may minimize this limitation. In addition, the calculation of the Cronbach’s alpha coefficient indicated good or very good reliability and consistency of the instruments used.

## Conclusions

The prevalence depression, anxiety and stress symptoms in the surveyed undergraduates was considered high and worrying, mainly due to the fact that the pandemic crisis in the state of Parana and Brazil was aggravated in the months following the present survey, with an increase in diagnoses and deaths, in addition to collapses in the health system, indicating that the pandemic has an important impact on the mental health of Brazilian undergraduate students.

The data suggest that higher levels of satisfaction with life and dimensions of psychological well-being, as well as the use of appropriate coping strategies, may constitute protective factors in undergraduates in relation to depression, anxiety and stress symptoms.

The study indicates the impact of the pandemic on the mental health of university students and suggests that educational actions that assist in the adoption of effective copings and promote conditions capable of increasing psychological well-being and satisfaction with life may be proposed as strategies to mitigate the psychological effects of present and future pandemic crises in this population.
